# Supplementary material for: Validation of a clinical and genetic model for predicting severe COVID-19
Source: Epidemiol Infect. 2022 Apr 25;150:e91. doi: 10.1017/S0950268822000541 (PMC9096108; doi:10.1017/S0950268822000541)
Supplement: Supplementary file 1 [file S0950268822000541sup001.docx]

Validation of a clinical and genetic model for predicting severe COVID-19

Gillian S. Dite, Nicholas M. Murphy, Erika Spaeth, Richard Allman, Lifelines Corona Research Initiative

# Supplementary information

**Supplementary Table S1.** Beta coefficients for calculation of risk of severe COVID-19

| **Variable** | **Value** | **Beta coefficient** |
| --- | --- | --- |
| Intercept |  | −1.37 |
| Age 18–29 years | 0 = no, 1 = yes | −1.31 |
| Age 30–39 years | 0 = no, 1 = yes | −0.83 |
| Age 40–49 years | 0 = no, 1 = yes | −0.40 |
| Age 50–69 years | 0 = no, 1 = yes | 0.00 |
| Age 70–74 years | 0 = no, 1 = yes | 0.57 |
| Age 75–79 years | 0 = no, 1 = yes | 0.82 |
| Age 80+ years | 0 = no, 1 = yes | 1.01 |
| Male | 0 = no, 1 = yes | 0.24 |
| Inverse of BMI | 10/BMI | −1.60 |
| Cancer, haematological | 0 = no, 1 = yes | 1.00 |
| Cancer, non-haematological | 0 = no, 1 = yes | 0.26 |
| Cerebrovascular disease | 0 = no, 1 = yes | 0.40 |
| Diabetes | 0 = no, 1 = yes | 0.43 |
| Hypertension | 0 = no, 1 = yes | 0.29 |
| Kidney disease | 0 = no, 1 = yes | 0.69 |
| Respiratory disease (excluding asthma) | 0 = no, 1 = yes | 1.17 |
| rs112317747 | 0 = T/T, 1 = C/T, 2 = C/C | 0.27 |
| rs2034831 | 0 = A/A, 1 = C/A, 2 = C/C | 0.24 |
| rs112641600 | 0 = C/C, 1 = T/C, 2 = T/T | −0.24 |
| rs10755709 | 0 = A/A, 1 = G/A, 2 = G/G | 0.12 |
| rs118072448 | 0 = T/T, 1 = C/T, 2 = C/C | −0.20 |
| rs7027911 | 0 = G/G, 1 = A/G, 2 = A/A | 0.10 |
| rs71481792 | 0 = A/A, 1 = T/A, 2 = T/T | −0.11 |

Note: Body mass index (BMI) is calculated as kg/m^2^; the inverse of BMI is calculated as 10 divided by BMI. In the current analysis, we used rs10905502 as a proxy for rs71481792 and rs78654835 as a proxy for rs112317747.

**Supplementary Figure S1.** Distribution of probability of severe COVID-19 in (A) all Lifelines COVID-19 cohort participants, (B) hospitalized (cases), (C) non-hospitalized confirmed COVID-19 (controls) and (D) non-hospitalized confirmed and self-reported COVID-19 (controls).

**Supplementary Figure S2.** Calibration plots for (A) the confirmed COVID-19 group and (B) the confirmed and self-reported COVID-19 group.
